# Relationship Satisfaction in People with Parkinson’s Disease and Their Caregivers: A Cross-Sectional Observational Study

**DOI:** 10.3390/brainsci11060822

**Published:** 2021-06-21

**Authors:** Johanne Heine, Hannah von Eichel, Selma Staege, Günter U. Höglinger, Florian Wegner, Martin Klietz

**Affiliations:** 1 Department of Neurology, Hannover Medical School, Carl-Neuberg-Straße 1, 30625 Hannover, Germany; heine.johanne@mh-hannover.de (J.H.); Hannah.C.V.Eichel@stud.mh-hannover.de (H.v.E.); Staege.Selma@mh-hannover.de (S.S.); Hoeglinger.Guenter@mh-hannover.de (G.U.H.); Wegner.Florian@mh-hannover.de (F.W.); 2 Center for Systems Neuroscience, Hannover Medical School, 30625 Hannover, Germany

**Keywords:** Parkinson’s disease, caregiver burden, relationship satisfaction, health-related quality of life

## Abstract

Parkinson’s disease (PD) is a neurodegenerative disorder, which leads to reduced health-related quality of life (HR-QoL) and autonomy in advanced stages of the disease. Hence, people with PD (PwPD) are in need of help, which is often provided by informal caregivers, especially spouses. This might influence the relationship satisfaction in patients and their spousal caregivers. Additionally, previous studies have shown that a reduced relationship satisfaction may result in mental disorders and reduced physical health. The aim of this study is to identify factors influencing PwPD and their caregivers’ relationship satisfaction in a cross-sectional observational study. Analyses revealed an overall satisfying relationship, measured by the Quality of Marriage Index, in PwPD (*n* = 84) and their caregivers (*n* = 79). Relationship satisfaction in PwPD mildly decreased with reduced HR-QoL and more severe depressive symptoms. Reduced relationship satisfaction in caregivers was significantly associated with decreased HR-QoL, higher caregiver burden, more severe depressive symptoms and increased neuropsychiatric symptoms in PwPD. Further studies are needed to investigate the influence of the identified factors over time and if relationship satisfaction has a reciprocal impact on caregiver burden, HR-QoL as well as mental and physical health.

## 1. Introduction

Parkinson’s disease (PD) is the second most common neurodegenerative disorder with an estimated prevalence of 0.5% of the population in Germany [1]. PD is characterized by the cardinal motor symptoms rigidity, tremor at rest, bradykinesia and postural instability. Further, non-motor symptoms such as cognitive impairment, depression, anxiety, impulse control disorders, obstipation and urinary urge-incontinence play a role in PD [2,3]. Due to the progressive nature of motor and non-motor symptoms in people with PD (PwPD), patients’ health-related quality of life (HR-QoL) and autonomy is reduced in advanced stages of the disease and patients are in need of help, which is often provided by informal caregivers, especially spouses [3,4,5,6,7].

For instance, already in early stages of the disease PwPD often experience a lack of fine motor skills, which increases with disease progression leading to the disability of fine motor skills to button a shirt or prepare food [8]. Furthermore, due to gait disturbances, PwPD tend to fall, often resulting in hospitalization [9] and the need of intensified care by the caregiver afterwards. With advanced stages of the disease, increased cognitive impairment additionally contributes to the need of help. In the mild cognitive impairment stage, independence of PwPD is often preserved in the majority of daily activities. However, PwPD may develop dementia during advanced stages of PD and finally lose their ability to take care of their finances and paperwork. Additionally, medication adherence and accuracy are reduced with increased cognitive impairment of the patient. Therefore, spousal caregivers take over these obligations in addition to their own responsibilities, resulting in less time for themselves. As a consequence, spousal caregivers may develop feelings such as fatigue, frustration and distress as well as fear regarding the future when thinking about further disease progression [10,11]. Hence, taking care of a patient with PD has been considered as leading to physical and psychological distress in caregivers, resulting in reduced quality of life and increased caregiver burden [3,12,13,14,15]. Furthermore, despite spending more time together due to care responsibilities of spousal partners, patients and caregivers feel emotionally more distant [11]. Reasons might be reduced communication and fewer conversations due to cognitive impairment of the patient [11]. Furthermore, previous studies showed that alexithymia, the disability of recognition and feelings of emotions, is markedly increased in PwPD, which might lead to the fact that PwPD neglect their caregivers’ negative feelings and burden [16,17].

Therefore, providing and receiving care may have an impact on the relationship satisfaction of patients with PD and their spousal caregivers. Previous studies showed an association between later disease stages of PD and spousal caregivers’ relationship satisfaction. Furthermore, especially non-motor symptoms have been found to influence caregivers’ relationship satisfaction in a negative way as well as increase caregiver burden and HR-QoL in caregivers [11,18,19]. Additionally, neurodegenerative diseases like PD and Alzheimer’s disease may result in the disability to manage difficult life situations as partners and therefore leading to higher caregiver burden, lower relationship satisfaction and more depressive symptoms in patients and spousal caregivers as reported in former studies [20]. However, the relationship between depressive symptoms and relationship satisfaction in PwPD and spousal caregivers has not been fully understood yet since diverse results in the past were reported [21,22]. Nonetheless, previous studies showed that a decreased relationship quality may result in mental disorders and reduced physical health leading to higher costs for individuals and society [23,24,25].

This study aims to identify factors influencing the relationship satisfaction of PwPD and their caregivers in a cross-sectional observational study.

## 2. Materials and Methods

### 2.1. Participants

Ethical approval for this study was provided by the local Ethics Committee of Hannover Medical School (No. 3178-2016, Amendment in 2018). Our sample included 84 PwPD and their 79 primary caregivers. Numbers of patient and caregiving participants differ since some of the caregivers denied the participation in this study. There were no significant differences between the PwPD characteristics between participants and the PwPD of the five spousal caregivers who refused study inclusion. All participants gave their written informed consent. PwPD included in the study had been neurologically diagnosed with PD for at least one year and met the Movement Disorder Society (MDS) clinical diagnostic criteria for PD [26].

The questionnaires were sent to the participants in January 2020 and returned until the end of March 2020 at the latest. Completing all questionnaires took approximately 45–60 min.

PwPD who lived in an institutional care facility, who had been diagnosed with atypical Parkinsonism and/or did not have an informal caregiver were excluded from this study. Only patients and their corresponding spousal caregivers were included in this study. Two of the relationships were homosexual relationships whereas in all remaining cases the relationships were heterosexual. Professional caregivers as well as informal but non-spousal caregivers (e.g., children) and their corresponding patients were excluded. Furthermore, the patient and the spousal caregiver were considered in this study only if the spousal caregiver was the primary caregiver and therefore spending the most time with the patient and managing most of the caregiving. Professional caregivers were excluded. Participants did not receive financial compensation for participating in this study.

### 2.2. Measures

To classify the patients’ PD impairment, Hoehn and Yahr staging was performed, which ranges from a minimum of one point (unilateral symptoms) to a maximum of five points (confinement to bed or wheelchair) [27].

Patients were asked to fill out the Parkinson’s disease quality of life questionnaire (PDQ-8) to assess their health-related quality of life (HR-QoL). The PDQ-8 is an excellently validated standard measure for HR-QoL in PD showing a high internal consistency with a Cronbach’s alpha of 0.59–0.79 as confirmed in former studies [28]. The questionnaire estimates the patient’s functioning and well-being and is composed of eight questions in eight domains on a five-point Likert scale ranging from 0 to 4 points (the higher the value, the lower the HR-QoL). The total score, ranging from 0 to 32 points, is converted in percentage representing the HR-QoL restrictions. A higher percentage displays a worse HR-QoL of the patient [29]. Caregivers were requested to help patients with disease-related impairment in completing the PDQ-8 to ensure correct results and avert anosognosia (as described in [3,18,30]).

To further investigate the impairment of daily living, patients were asked to perform the Movement Disorder Society Unified Parkinson’s Disease Rating Scale (MDS-UPDRS) part I and II, which is the most established scale for measurement of PD symptoms with high internal consistency and a Cronbach’s alpha of 0.79–0.93 across parts. Furthermore, the MDS-UPDRS correlated with the original UPDRS in former studies [31]. Part I of the MDS-UPDRS involves non-motor aspects of experiences of daily living whereas part II encompasses motor aspects of experiences of daily living. Both parts consist of 13 items on a five-point Likert scale from 0 (no symptoms) to 4 (severe symptoms). Thus, a patient can reach a maximum of 52 points in each part, with 52 points indicating the worst extent of symptoms [31]. Neuropsychiatric symptoms in patients were determined by using the checklist for the detection of neuropsychiatric disorders in Parkinson’s disease (CEND-PD). The German version of the CEND-PD has been found to be sufficiently reliable and valid [32] and includes 12 items, which are divided into three subscales (psychotic symptoms, mood/apathy, disturbance of impulse control). The questions can be answered on a five-point Likert scale from 0 (no symptoms) to 4 (severe symptoms), each subscale has a maximum of 20 points. A neuropsychiatric condition is assumed if 3 points out of 20 have been reported [32].

Beck’s depression inventory (BDI) was used to determine patients’ and caregivers’ depressive mood (depressive symptoms ranging from 0–63 points: 0–13 no depression, 14–19 mild, 20–28 moderate, 29–63 severe depression) [33]. The BDI is one of the standard measures to estimate the severity of depression with good reliability quantified in various studies (for review, see [34]). Furthermore, the BDI has been described as a reliable and valid instrument to assess depressive symptoms in the setting of PD [35].

In addition, caregivers were asked to answer the short form 36 health survey (SF-36) to measure their HR-QoL [36]. This questionnaire is one of the gold standard instruments to assess the HR-QoL showing good reliability of the items with a Cronbach’s alpha > 0.80 and reliability of >0.90 compared to other measures of quality of life [37]. The SF-36 includes eight dimensions. Subscale scores were percentage-transformed. Since all subscale scores were highly correlated, an average score across the eight scales was calculated as done in previous work with our group [30]. As a result, a score of 0 indicates a maximum of impairment whereas a score of 100 indicates the absence of impairment.

In order to assess caregiver burden, caregivers were provided with the recently developed and validated German version of the Parkinson’s disease caregiver burden questionnaire (PDCB). In the English version of the PDCB, the PDCB correlated significantly with the Caregiver Burden Inventory as a general caregiver burden questionnaire showing a Cronbach’s alpha of 0.856 [38]. In the validated German version of the PDCB, a significant correlation with the Zarit Burden Interview-22 was found (*r* = 0.71; *p* < 0.001 as a general caregiver questionnaire and total SF-36 score of the caregiver *r* = −0.40; *p* = 0.001) [30,39]. The first part of the PDCB consists of 20 items on a five-point Likert scale (0 to 4). Therefore, participants can reach a maximum of 80 points on this part of the PDCB. Additionally, caregivers were requested to estimate their global burden as a caregiver on a scale from 0 to 100. For the total caregiver burden score, points from the first part and 20% of the estimated global burden were summed up. The total PDCB score can range from 0 to 100 with higher scores displaying higher caregiver burden [30,38].

To assess the quality of their relationship, patients and caregivers were asked to complete the German version of the Quality of Marriage Index (QMI). The questionnaire showed high reliability with a Cronbach’s alpha of 0.94 in a recently published study [23] and contains six items. Five items can be answered on a seven-point Likert scale from 1 (very strong disapproval) to 7 (very strong approval) whereas one item can be answered on a ten-point Likert scale from 1 (very unhappy) to 10 (perfectly happy). For the total QMI-score, all points are summed up. The score can range from 6 to 45 points with higher scores indicating a higher quality of the relationship. A cut-off score of 34 or higher defines individuals as being satisfied with their relationship [23].

Patients and caregivers were also requested to provide general information about their background and demographics.

### 2.3. Analyses

Linear regression analyses was used to examine potential predictors of relationship satisfaction (QMI score) in PwPD and their spousal caregivers. Generally, a *p* value of ≤0.05 was considered as significant. For all statistical analyses, the level of significance was set to 0.05/number of analyses to adjust for multiple testing. To compare age, BDI and QMI scores in patients and caregivers a test for normal distribution and afterwards an unpaired *t*-test or Mann–Whitney Test was performed. Data were displayed in mean ± standard deviation (SD) as well as minimum (min) and maximum (max) for descriptive analyses. Statistical analyses were carried out using SPSS 25.0 (IBM, Armonk, NY, USA). Due to the exploratory nature of the study, no sample size calculation was performed.

## 3. Results

### 3.1. Patient and Caregiver Characteristics

Demographic and clinical characteristics of PD patients (*n* = 84, 30 females) and caregivers (*n* = 79, 50 females) are shown in Table 1. On average, patients were 68.4 years old (±9.9; min 41; max 88) with a disease duration of 10.5 years (±6.4; min 1; max 29). With regard to non-motor and motor aspects of experiences of daily living, evaluated by the MDS-UPDRS I and MDS-UPDRS II, patients scored 9.9 points (±5.2; min 1; max 27) and 17.8 (±10.7; min 4; max 48), respectively. PDQ-8 displayed an average of 35.1 points (±18.1; min 3.1; max 84.4), indicating moderate HR-QoL restrictions of the patients in general. On average, there were no to mild depressive symptoms in PD patients with a mean score of 12.3 on BDI (±8.1; min 0; max 48). The majority of 52 patients showed no or very low symptoms of depression whereas 12 patients suffered from mild and and 15 patients from moderate symptoms of depression, respectively. Three patients showed severe symptoms of depression. Neuropsychiatric symptoms, evaluated by CENS-PD, were also low to mild with an average of 7.2 points (±6.8; min 0; max 36). Regarding the relationship satisfaction, patients achieved 37.7 points on the QMI (±7.4; min 14; max 45) with 79.2% of patients having a QMI score of 34 points or higher, suggesting an overall satisfying relationship with their caregiving spouse.

Caregivers were 67.2 years old (±9.9; min 37; max 88), spending an average of 5.8 h per day (±6.6; min 0; max 24) with caregiving. Overall, caregivers scored 31.4 points on PDCB (±16.1; min 0; max 79) indicating a mild to moderate caregiver burden. The caregivers’ HR-QoL, estimated by the SF-36, was moderate with an average score of 63.8 (±19.7; min 16.4; max 92.9). With regard to depressive symptoms BDI displayed an average of 9.2 points (±6.7; min 0; max 28), suggesting no or very low depressive symptoms. The majority of 62 caregivers showed no or very low depressive symptoms while 10 and 8 caregivers displayed mild or moderate symptoms of depression, respectively. In addition, BDI scores were significantly lower in caregivers (9.2 ± 6.7) compared to PD patients (12.3 ± 8.1; *p* = 0.009). In the QMI 71.2% of the caregivers reached 34 points or higher. The mean QMI score was 36.1 points (±9.2; min 9; max 45) indicating an overall satisfying relationship comparable to patients’ relationship satisfaction. There was no significant difference between PD patient and caregiver QMI score detectable (*p* = 0.46).

### 3.2. Factors Influencing Patients’ and Caregivers’ Relationship Satisfaction

Factors investigated with regard to relationship satisfaction in patients and caregivers are displayed in Table 2 and Table 3, respectively. Analyses revealed a correlation between PDQ-8 scores and QMI scores of PD patients, suggesting that relationship satisfaction decreased with higher PDQ-8 scores and therefore with lower HR-QoL. Furthermore, analyses displayed that patients’ relationship satisfaction was also reduced with increasing BDI scores. Hence, more severe depressive symptoms may be a predictor for developing a decreased relationship satisfaction. After correction for multiple testing, no significant correlations between the examined factors and relationship satisfaction in PD patients could be found (Table 2).

With regard to caregivers’ relationship satisfaction, higher HR-QoL of caregivers, estimated by the SF-36, is significantly associated with higher relationship satisfaction.

In addition, analyses revealed a significant correlation between BDI scores, CENS-PD and PDCB scores with relationship satisfaction indicating that more severe depressive symptoms in caregivers, more neuropsychiatric symptoms in patients as well as higher caregiver burden are associated with a decreased quality of the relationship in caregivers. These factors remained significant after correction for multiple comparisons in the linear regression analysis of caregivers’ QMI (Table 3).

Regarding the caregivers’ gender, a tendency to a reduced relationship satisfaction in male caregivers could be detected even though no significant association could be found after correction for multiple testing.

Age, disease duration, MDS-UPDRS I and II of the patient and caregiving hours per day were not significantly associated with the relationship satisfaction in spousal caregivers.

## 4. Discussion

In the present cross-sectional observational study, relationship satisfaction in a German cohort of PwPD and their caregivers was assessed. In advanced stages of the disease, patients suffer from a decreased HR-QoL and reduced autonomy due to exacerbated motor and non-motor symptoms. Therefore, patients are in need of support regarding activities of daily living. In many cases, the support is provided by informal caregivers, like family members and especially spouses [7]. One can assume that taking care of a patient with PD has an impact on the relationship of PwPD and their spousal caregivers. Despite spending more time together due to care responsibilities, the intimate side of the relationship might get lost since spousal caregivers and patients predominantly focus on the disease and disease related symptoms instead of the emotional aspect of their relationship.

Previous studies showed an association between relationship satisfaction and well-being. Furthermore, a correlation between decreased relationship quality and mental disorders and reduced physical health was detected in former studies, which might lead to higher costs for individuals and society, e.g., the health care system [22,23,24,35]. Thus, the detection of relationship dissatisfaction as well as the mental and physical health status seems to be necessary to further address and investigate this relationship and develop interventions. Recently, a German version of the Quality of Marriage Index to investigate the relationship satisfaction was developed and validated in a German cohort [22].

However, only a few studies have addressed the topic of relationship satisfaction in people with PD and their spousal caregivers so far.

Champagne et al. found no significant difference concerning the relationship satisfaction in PwPD compared to their caregivers [40]. This is in line with previous investigations in which patients with young-onset PD and their partners did not show a significant difference in their relationship satisfaction [21]. Likewise, we found a comparable relationship satisfaction in patients and caregivers. In contrast to these findings, Ricciardi et al. reported a reduced quality and satisfied relationship in fifteen PwPD compared to their partners [22].

With regard to the factors influencing the relationship satisfaction in PwPD, more severe depressive symptoms in PwPD, represented by higher BDI scores, were associated with a lower relationship satisfaction in our study. Moreover, patients had significantly increased depressive symptoms compared to caregivers. This is in line with previous studies, in which PwPD showed more severe depressive symptoms compared to their partners or caregivers [21,41] and the severity of depressive symptoms seemed to have a negative influence on the relationship satisfaction [21]. In contrast, Ricciardi et al. described no influence of depressive symptoms on the relationship satisfaction in PwPD and their caregivers [22]. Explanations might be due to the smaller cohort of 15 PwPD and spousal caregivers in the study by Ricciardi et al. as well as the use of a different assessment (Hamilton Depression Rating Scale) to detect depressive symptoms.

In our study, a decreased patient relationship satisfaction was associated with a reduced HR-QoL in the PDQ-8. According to prior research, reduced HR-QoL is influenced by motor and non-motor symptoms such as depression, anxiety, executive and attentional cognitive deficits as well as REM sleep behavior disorder and impulse control disorders [6,18,42]. Moreover, a combination of motor and non-motor experiences of daily living was found to be tightly related with HR-QoL [43]. Interestingly, non-motor and motor-impairment in daily living of the patient, represented in part I and II of the MDS-UPDRS, did not directly influence the relationship satisfaction in PwPD in this cohort. To the best of our knowledge, this is the first study investigating the association between part I and II of the MDS-UPDRS and relationship satisfaction in PwPD.

Further studies have reported that female PwPD suffer from non-motor functions, including fatigue and depression, in a more severe way compared to male PwPD [44]. Additionally, female sex in PwPD was found to be a predictor for a worse HR-QoL in the two subdomains, physical function and socio-emotional QoL [45]. Male PwPD instead were more afflicted by cognitive deficits in HR-QoL [45]. Thus, it could be assumed that the gender might have an influence on the relationship satisfaction. Interestingly, no significant association between the patients’ gender and relationship satisfaction was found in our cohort. This is in contrast with previous studies, which reported a lower relationship satisfaction in females [23,46]. However, these studies did not focus on the participants’ health status and there is limited information about gender specific relationship satisfaction in PwPD.

In the present study, an association between male sex in caregivers and a reduced relationship satisfaction could be detected. As reported by Faulkner et al., a greater wife’s well-being leads to a greater husband’s marital satisfaction in an American cohort [47]. Additionally, Faulkner et al. detected wives’ depression as a predictor for a decrease in husbands’ marital satisfaction [47]. As non-motor symptoms like depression are more present in female PwPD, one may speculate that an impaired health status along with increased depressive symptoms in the patient with PD has a greater impact on the relationship satisfaction of male caregivers in our cohort. Faulkner et al. found a change in the husband’s physical health being also a predictor for a decreased wife’s satisfaction over time and assumed the additional task of taking care of the partner leads to more burden in the wife. However, the study was not specific for PD [47]. Nevertheless, in recent PD specific studies, higher caregiver burden had been described in female caregivers compared to male caregivers [48,49,50]. Contrary, Lee et al. reported higher caregiver burden in male caregivers of PwPD while Sanyal et al. could not detect gender specific differences in caregiver burden in a community-based study from India [51,52]. In former investigations in our cohort also no significant difference in gender specific caregiver burden could be identified, suggesting that the gender does not significantly impact burden [41] whereas relationship satisfaction in caregivers seems to be influenced by gender in our cohort.

Disease duration as well as the patient’s PD stage and impairment, represented by the Hoehn and Yahr score, did not influence the relationship satisfaction in the present study. This is consistent with previous findings in other PD cohorts [22].

Taking care of a patient with PD is a responsibility that is often fulfilled by patients’ spouses who act as informal caregivers. Former studies have shown that this role may result in higher levels of distress and decreased quality of life [18,53,54] and has a negative impact on emotional well-being in spousal caregivers [55] resulting in caregiver burden [56,57].

Vatter et al. investigated the relationship satisfaction in male PwPD with cognitive impairment and their female spousal caregivers. The spouses’ satisfaction of the relationship had decreased with later stages of the husband’s disease. Even though patients and their partners spent more time together in order to allow spousal caregivers to support their husbands in managing daily activities, the spouses felt more disconnected and emotionally distant to their partners [22]. Furthermore, with a higher stage of the disease and appearance of dementia, spouses developed feelings of loneliness due to lack of communication with their partners. In our cohort, we did not find a significant association between the caregiving hours and relationship satisfaction. Therefore, in synopsis with the afore-reported findings, one can hypothesize that not the quantity but the quality of the time spent together may have an influence on the relationship satisfaction.

A study by Modugno et al. identified caregiver change in the ability to perform family duties and leisure activities due to care responsibilities to be one of the main influencing factors on caregiver burden [20]. This is in line with another study in patients with Alzheimer’s disease or PD and their spousal caregivers, which reported loss of the ability to work as partners to manage difficult life situations, loss of shared identity and view resulted in higher caregiver burden, more depressive symptoms and lower relationship satisfaction [58].

In our present study, we found an overall satisfying relationship in spousal caregivers. However, higher caregiver burden (PDCB) was associated with decreased QMI scores indicating a reduced relationship satisfaction in spousal caregivers. In addition to the factors contributing to caregiver burden mentioned above, motor and non-motor symptoms in PwPD including psychosis, depression, impulse control and sleep disorders are related to higher caregiver burden [18,19,56] and may therefore result in decreased relationship satisfaction. In line with this, more severe neuropsychiatric symptoms in PwPD (CEND-PD) were significantly associated with a decreased relationship satisfaction in our cohort of caregivers.

Moreover, a previous study in PwPD and their spousal caregivers from Mexico has suggested that greater motor and non-motor-impairments in PwPD predict a higher caregiver burden resulting in greater mental health deficits in caregivers [59]. Furthermore, caregivers of PwPD present higher depression scores compared to non-caregiving spouses [57]. Our study showed that severe depressive symptoms (BDI) were associated with a reduced relationship satisfaction in caregivers. Additionally, depression was found to be an affecting factor on caregivers HR-QoL [57]. In our cohort, a lower HR-QoL (SF-36) in caregivers was associated with lower relationship satisfaction. Therefore, depressive symptoms and lower quality of life may have an important impact on the relationship satisfaction in PD caregivers.

Finally, interventions to improve relationship satisfaction and decrease caregiver burden are needed in PD therapy. One may speculate that mindfulness interventions could reduce caregiver burden and improve relationship satisfaction [60]. This hypothesis has to be tested in future studies.

### Limitations

First, we focused on the current status of the relationship and did not consider the quality of the relationship before the diagnosis of PD, which might contribute to the current relationship satisfaction or dissatisfaction. Furthermore, we did not take other aspects into consideration which might influence the quality of the relationship (e.g., comorbidities of our patients, relationship duration, financial burden, relationship therapy).

Moreover, the participants in our study had an overall satisfying relationship as well as moderate restrictions in HR-QoL and mild to moderate caregiver burden. Unfortunately, severely affected patients and burdened caregivers denied the participation in this study, as described in former studies of our group [41]. Therefore, the quality of relationship in severely affected patients and burdened caregivers remains unclear.

Another restriction was the cross-sectional structure of this study. Longitudinal data would be desirable to find out about the dynamics in the relationship satisfaction and the influence of the investigated factors on the relationship quality over time.

Due to the exploratory mono-centric observational design of the study, no pre-registration was performed.

## 5. Conclusions

Thus far only a few studies have addressed the relationship satisfaction in patients with PD and their informal caregivers. This study found a comparable relationship satisfaction in PD patients and their spousal caregivers with an overall satisfying relationship. Factors negatively influencing the relationship satisfaction in PD patients were depressive symptoms and HR-QoL. In spousal caregivers, decreased relationship satisfaction was significantly associated with severity of depressive symptoms, reduced HR-QoL, neuropsychiatric symptoms in PD patients and caregiver burden. Further studies are needed to investigate the contribution of these factors to the relationship satisfaction over time and to examine a possible reciprocal influence of relationship satisfaction on caregiver burden, HR-QoL, as well as mental and physical health.

## Figures and Tables

**Table 1:** Patient (*n* = 84, *n* = 30 females) and caregiver (*n* = 79, *n* = 50 females) characteristics.

	Mean ± SD	Min	Max
PwPD			
Age (years)	68.4 ± 9.9	41	88
Disease duration (years)	10.5 ± 6.4	1	29
PDQ-8	35.1 ± 18.1	3.1	84.4
H&Y	2.9 ± 1.0	1	5
MDS-UPDRS I	9.9 ± 5.2	1	27
MDS-UPDRS II	17.8 ± 10.7	4	48
BDI	12.3 ± 8.1	0	48
QMI	37.7 ± 7.4	14	45
CENS-PD	7.2 ± 6.8	0	36
Caregivers			
Age (years)	67.2 ± 9.9	37	88
Caregiving h/day	5.8 ± 6.6	0	24
SF-36	63.8 ± 19.7	16.4	92.9
PDCB total	31.4 ± 16.1	0	79
BDI	9.2 ± 6.7	0	28
QMI	36.1 ± 9.3	9	45

BDI, Beck’s depression inventory; CENS-PD, checklist for the detection of neuropsychiatric disorders in Parkinson’s disease; Max, Maximum; Min, Minimum; MDS-UPDRS, Movement Disorder Society Unified Parkinson’s Disease Rating Scale; PD, Parkinson’s disease; PDCB, Parkinson’s disease caregiver burden questionnaire; PDQ-8, Parkinson’s disease questionnaire 8; SD, standard deviation; SF-36, short form 36 health survey; QMI, Quality of Marriage Index.

**Table 2:** Linear regression analysis of factors influencing patients’ Quality of Marriage Index (QMI).

PwPD’ Variables	*R* ^2^	Beta	*p*
Sex (female 1, male 2)	0.031	−0.175	0.115
Age (years)	0.013	0.115	0.302
Disease duration (years)	0.013	−0.115	0.340
PDQ-8	0.057	−0.238	0.032 *
H&Y	0.004	−0.060	0.605
MDS-UPDRS I	0.025	−0.157	0.164
MDS-UPDRS II	0.022	−1.49	0.183
BDI	0.070	−0.265	0.017 *

Linear regression analyses of factors influencing patients’ relationship satisfaction. *p* value adjustment for multiple testing was 0.05/8 = 0.0063. * Significant correlation at *p* ≤ 0.05. BDI, Beck’s depression inventory; Hoehn and Yahr; MDS-UPDRS, Movement Disorders Society Unified Parkinson’s Disease Rating Scale; PDQ-8, Parkinson’s disease questionnaire 8. A correlation between PDQ-8 and BDI scores with relationship satisfaction in PD patients could be detected but after correction for multiple testing no significant correlations between the examined factors and relationship satisfaction in PD patients could be found.

**Table 3:** Linear regression analysis of factors influencing caregivers’ Quality of Marriage index (QMI).

Variables	*R* ^2^	Beta	*p*
Sex (female 1, male 2)	0.058	0.240	0.037 *
Age (years)	0.043	0.207	0.073
Patients’ disease duration (years)	0.0001	−0.012	0.924
Caregiving h/day	0.005	0.071	0.560
SF-36	0.101	0.318	0.005 ***
PDCB total	0.450	−0.671	<0.001 ***
BDI	0.348	−0.590	<0.001 ***
CENS-PD	0.341	−0.584	<0.001 ***
MDS-UPDRS I	0.015	−0.123	0.291
MDS-UPDRS II	0.011	−0.105	0.368

Linear regression analyses of factors influencing caregivers’ relationship satisfaction. *p* value adjustment for multiple testing was 0.05/10 = 0.005. * Significant correlation at *p* ≤ 0.05. *** Significance level at *p* ≤ 0.005. BDI, Beck’s depression inventory; CENS-PD, Checklist for the detection of neuropsychiatric disorders in Parkinson’s disease; PDCB, Parkinson’s disease caregiver burden questionnaire; SF-36, short form 36 health survey. SF-36, PDCB, BDI and CENS-PD showed an association with relationship satisfaction in caregivers. These factors remained significant after correction for multiple comparisons. A tendency to a reduced relationship satisfaction in male caregivers could be detected even though no significant association could be found after correction for multiple testing.

## Data Availability

Data are available on reasonable request to the corresponding author M.K.
